# Assessing Seasonal Nitrate Contamination by Nitrate Dual Isotopes in a Monsoon-Controlled Bay with Intensive Human Activities in South China

**DOI:** 10.3390/ijerph17061921

**Published:** 2020-03-16

**Authors:** Jiacheng Li, Ruixue Cao, Qibin Lao, Fajin Chen, Chunqing Chen, Xin Zhou, Yafei Meng, Qingmei Zhu

**Affiliations:** 1 College of Ocean and Meteorology, Guangdong Ocean University, Zhanjiang 524088, China; jcli1209@163.com (J.L.); cao_ruixue@163.com (R.C.); laoqibin@163.com (Q.L.); chenchunqing1221@163.com (C.C.); zhouxin_hc1993@163.com (X.Z.); mengyafei199001@sina.com (Y.M.); zhuqingmei1987@163.com (Q.Z.); 2 Marine Environmental Monitoring Centre of Beihai, State Oceanic Administration, Beihai 536000, China

**Keywords:** nitrate dual isotopes, nitrate sources, Bayesian isotope mixing model, Zhanjiang Bay

## Abstract

Nitrate (NO_3_^−^) dual isotope analysis was performed in Zhanjiang Bay, which is a closed bay with intensive human activities in South China, to investigate seasonal changes in the main NO_3_^−^ sources and their biogeochemical processes in the monsoon-controlled climate. The relatively low N/P ratios in Zhanjiang Bay suggests that nitrogen (N) is a limiting nutrient, which indicates that the increase of N is favorable for phytoplankton proliferation. However, a sufficient amount of ammonium was found in our study area owing to intensive human activities, which can support biological processes. Thus, less NO_3_^−^ biological processes were found, indicating that NO_3_^−^ isotopic characteristics may reveal details of the mixing from various sources. The Bayesian mixing model showed that NO_3_^−^ in the upper bay originated from manure (43%), soil N (30%), N fertilizer (17%), and N precipitation (10%) during winter, which reflects the local human activities; while NO_3_^-^ sources during summer were mainly N fertilizer (36%), soil N (32%), and manure (31%), indicating the source as the runoff from the upper river basin. Our results suggest that nitrate dual-isotope was very useful for tracing the main NO_3_^−^ sources in the condition of the sufficient ammonium, and runoff exerted an important impact on the shift in NO_3_^−^ sources between both the local source and the source from the upper river basin during the two seasons in this monsoon-controlled bay.

## 1. Introduction

Eutrophication in coastal aquatic ecosystems has prompted wide public concern owing to significantly increased nutrient inputs to coastal waters [1,2,3]. N loads to the coastal environment have been increasing as a result of intensive industrial activity, agricultural activity, and rapid urbanization in coastal areas [4,5,6,7]. Heavy nitrate (NO_3_^−^) pollution has risen above acceptable levels in many areas, which greatly alters the N cycle in aquatic ecosystems, thereby endangering coastal ecosystems and biogeochemical cycles [8,9,10]. Thus, it is necessary to trace N sources and determine its recycling processes in coastal areas. 

The traditional, and simplest method to determine the nitrate pollution source is to identify the pollution source by investigating the land use type of the pollution area and combining it with the analysis of hydrochemical characteristics. However, due to the diversity of nitrate sources, the mixing of point and nonpoint sources and the complex physical, chemical, and biological transformation processes in the nitrogen cycle, the results obtained by this traditional method are relatively rough. In contrast, since different NO_3_^−^ sources (e.g., manure and sewage, soil organic N, and fertilizer and atmospheric deposition) have distinct isotope ratios of NO_3_^−^ (δ^15^N-NO_3_^−^ and δ^18^O-NO_3_^−^) [4,6,11] and isotope ratios also reflect N biogeochemical processes [9,12,13,14], it is possible to identify these N sources using isotope fingerprints. For example, NO_3_^−^ that originates from domestic manure and sewage is more enriched in δ^15^N-NO_3_^−^ (10‰–20‰) than NO_3_^−^ that originates from atmospheric deposition and fertilizer because of the volatilization of heavy N isotope-depleted ammonia produced from animal and human waste. In addition, δ^18^O-NO_3_^−^ values, in atmospheric deposition, are generally very high (50‰–80‰) compared to those from other sources (<25‰) [6,15]. In addition, biological processes such as assimilation and denitrification can cause isotope fractionation owing to preferential uptake of lighter isotopes (^14^N and ^16^O) [9,13]. During phytoplankton assimilation and denitrification, the enrichment of isotope values with a ^15^N/^14^N:^18^O/^16^O ratio (^18^ε:^15^ε) of 1 occurs [12,13,16]. Nitrification results in the formation of ^15^N-depleted NO_3_^−^, and remineralization of sinking organic N produced by N_2_ fixation results in an increase in the proportion of light N in seawater [14]. Under the distinct isotope fingerprints of various N sources, a Bayesian stable isotope mixing model was successfully applied for NO_3_^−^ source identification [11,17]. Thus, a better understanding of N sources and its cycling in the coastal environment could be achieved through integrated knowledge of dual NO_3_^−^ isotope signatures. 

Zhanjiang Bay is a closed bay with intensive human activities in South China. Recently, high loads of inorganic N and phosphate (PO_4_^3−^) were found in the seawater of Zhanjiang Bay, owing to the rapid economic growth and urban development in the surrounding area [18]. Clear spatial and seasonal variations in the degree of eutrophication were previously reported in Zhanjiang Bay [19]. Particularly, significantly high nutrients were found in the upper bay area, and excess dissolved inorganic nitrogen (DIN) was the dominant factor for the eutrophication in the bay, which is considered to be greatly related to land-based pollution [19]. However, in a monsoon-controlled bay with such frequent and intensive human activities in South China, the seasonal variations in sources of NO_3_^−^ and its biogeochemical processes were still unclear. Thus, the study on the source of nutrients is of great significance to control the eutrophication of the regional water. The dual nitrate isotopes have proven useful in identifying nitrogen sources in aquatic ecosystems. Therefore, to investigate seasonal NO_3_^−^ sources and their biogeochemical processes, seasonal values of δ^15^N-NO_3_^−^ and δ^18^O-NO_3_^−^ were measured in Zhanjiang Bay in 2017, as were concentrations of nutrients (PO_4_^3-^), nitrite (NO_2_^−^), NO_3_^−^, and ammonium (NH_4_^+^) and physicochemical parameters. These data were used to determine the predominant sources of NO_3_^−^ and its biological transformation in the bay, as well as the controlling factors influencing the NO_3_^−^ distribution.

## 2. Materials and Methods

### 2.1. Field Sampling

**Figure 1:**
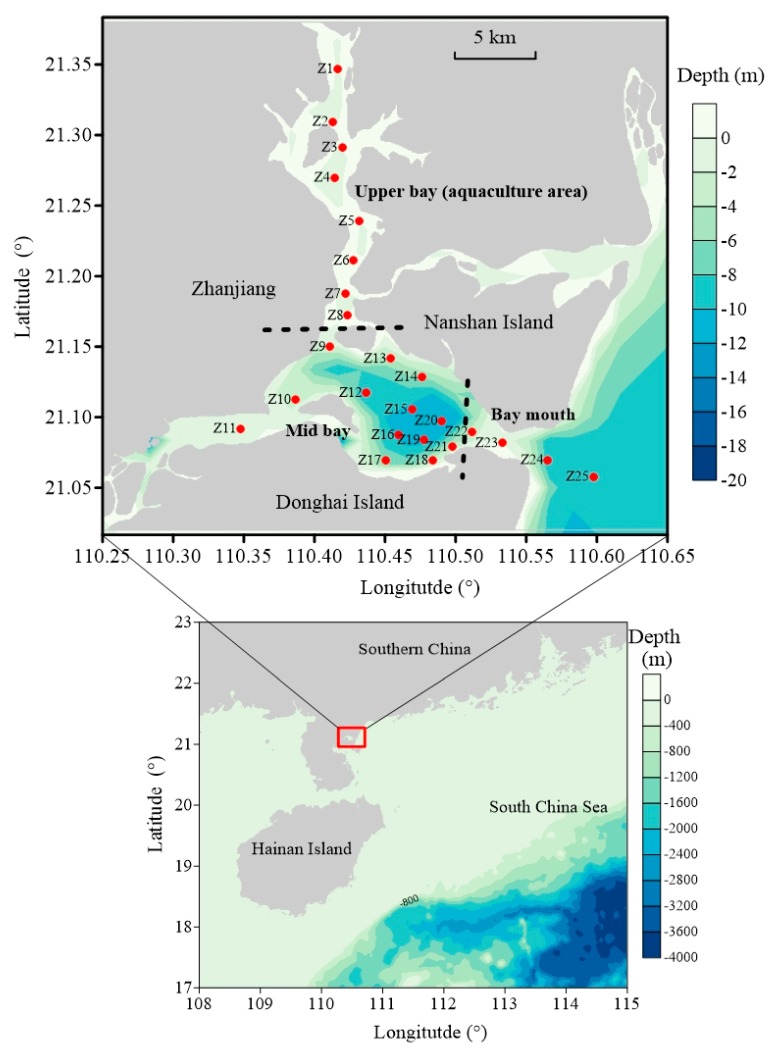
Map of Zhanjiang Bay showing the sampling stations. The red circle denotes the sampling station. The black dashed line indicates the boundary between the upper bay, mid-bay, and bay mouth segments. The upper bay is mainly used for aquaculture activities and the mid-bay is mainly used for shipping.

Zhanjiang Bay is a monsoon-controlled bay in the west of Guangdong Province, South China. It is a complex area—geographically and hydrodynamically speaking—and is significantly influenced by intensive human activities, such as mariculture, industry, agriculture, and shipping. Two water regimes, including local river discharge and oceanic water from the northern area of the South China Sea, significantly influence the water of Zhanjiang Bay (Figure 1). The Bay is a deep-water bay and is surrounded by Zhanjiang City. The width of the bay at the mouth is approximately 2 km. Since the mouth is narrow and shallow, it is difficult to discharge pollutants out to sea. As shown in Figure 1, the upper bay (station Z1) is subject to a high pollution burden and is used for oyster farming, while the mid-bay and bay mouth are mainly influenced by domestic sewage and nonpoint sources. Zhanjiang Bay is a monsoon-controlled bay with significant precipitation under southwest-heading monsoon in the spring and summer months. Heavy rainfall in the bay usually occurs in fall and summer (accounting for 75% of the yearly rainfall), while the dry season usually occurs in winter and spring [20]. Sampling was conducted in summer (June 2017) and winter (December 2017). Seawater samples were collected from 25 stations in the bay using a rosette sampler fitted with 12 L Niskin bottles (Figure 1). The eastern bay mouth was the main water exchange pathway, the western levee construction prohibited the water exchange in the western bay mouth; Figure 1 describes the seawards section that represents the directions from the upper-bay to the bay mouth.

### 2.2. Chemical Analysis

Salinity, temperature, and depth of water samples were measured using an RBR maestro multiparameter water quality monitor on-site. Except for the salinity and temperature, the other chemical parameter samples were only collected from surface water. Dissolved oxygen (DO) was determined using the Winkler titration method (Strickland and Parsons, 1972). The seawater was filtered using glass fiber filters (Whatman, 0.7 μm, GF/F) to determine Chlorophyll *a* (Chl *a*), and the filtered GF/F were stored at −20 °C before further processing and analysis. Nutrients and isotope samples were prefiltered through precombustion (450 °C for 4 h) GF/F membranes (47 mm diameter; Whatman) and stored at −20 °C until analysis.

NO_3_^−^ was determined by the cadmium–copper reduction method. Nutrients, including NO_3_^−^, NO_2_^−^, and PO_4_^3−^, were determined by a San^++^ continuous flow analyzer (Skalar, Netherlands). NH_4_^+^ concentrations were determined by spectrophotometry. 

For the determination of the NO_3_^−^ isotopes, NO_2_^−^ was removed by sulphamic acid, and the analysis of δ^15^N-NO_3_^−^ and δ^18^O-NO_3_^−^ followed the method modified from Mcllvin and Altabet [21]. According to this method, NO_3_^−^ was reduced to NO_2_^−^ by Cd and then further reduced to nitrous oxide by sodium azide in an acetic buffer (pH 4–5). After reduction, TraceGas (Isoprime) was used to separate and purify the nitrous oxide, and δ^15^N-NO_3_^−^ and δ^18^O-NO_3_^−^ were determined by Isoprime 1000. The standard deviations of δ^15^N-NO_3_^−^ and δ^18^O-NO_3_^−^ were less than 0.2‰ and 0.5‰, respectively. The isotopes of δ^15^N and δ^18^O were calibrated using international standard IAEA-N3. The reproducibility of duplicate analyses for δ^15^N-NO_3_^−^ and δ^18^O-NO_3_^−^ was less than 0.6‰ (average of ± 0.3‰) and 0.3‰ (average of ± 0.1‰), respectively.

### 2.3. Mixing Model

A salinity-based conservative mixing model was used to calculate the NO_3_^−^ concentrations and dual-isotope values of NO_3_^−^ from the simple physical mixing between freshwater and seawater endmembers [5,6,22,23]. The equations are as follows:

(1) $$q_{r}+q_{m}=1$$

(2) $$q_{r}S_{r}+q_{m}S_{m}=S_{mix}$$

(3) $$q_{r}N_{r}+q_{m}N_{m}=N_{mix}$$

(4) $$q_{r}N_{r}\delta_{r}+q_{m}N_{m}\delta_{m}=N_{mix}\delta_{mix}$$

where *q_r_* and *q_m_* represent the proportional contributions of freshwater and seawater, respectively. S, N, and δ represent salinity, the NO_3_^−^ level, and δ^15^N-NO_3_^−^ or δ^18^O-NO_3_^−^, respectively. S*_mix_*, N*_mix_*, and δ*_mix_* are mixtures of the two endmembers. Based on the above equations, the following equations can be obtained:

(5) $$q_{1}=\frac{S_{mix}-S_{2}}{S_{1}-S_{2}}$$

(6) $$\delta_{mix}=\frac{q_{1}\left(\delta_{1}N_{1}-\delta_{2}N_{2}\right)+\delta_{2}N_{2}}{N_{mix}}$$

Under steady-state conditions, the NO_3_^−^ concentration varied linearly along the mixing gradient, whereas the salinity-based isotopic mixing showed curvilinear behavior (Equation (6)) that reflected the concentration-weighted volumes of the two endmembers. 

### 2.4. SIAR Model

NO_3_^−^ sources in mixed samples were quantified using the Bayesian stable isotope mixing model. The model was conducted in SIAR (Stable Isotope Analysis in R, MixSIAR version 3.1.10, OmicX company, Le-Petit-Quevilly, France). The framework of this model is as follows:

(7) $$X_{ij}=\overset{k}{\underset{k=1}{\sum }}P_{k}\left(S_{jk}+c_{jk}\right)+\varepsilon_{ij}\;S_{jk}\sim N\;\left(\mu_{jk},\;\omega_{jk}^{2}\right)\;C_{jk}\sim N\;\left(\lambda_{jk},\;\tau_{jk}^{2}\right)\;\varepsilon_{jk}\sim N\;\left(0,\;\sigma_{j}^{2}\right)$$

where *X_ij_* represents the dual-isotope values of NO_3_^−^ (δ^15^N-NO_3_^−^ and δ^18^O-NO_3_^−^) of a mixed sample; *S_jk_* represents the isotope values of NO_3_^−^ sources normally distributed with an average of *μ_jk_* and standard deviation of *ω_jk_*; *P_k_* represents the proportional contributions of NO_3_^−^ sources; *c_jk_* represents the fractionation factor for the dual NO_3_^−^ isotopes on NO_3_^−^ sources, which is normally distributed with an average of λ*_jk_* and standard deviation of *τ_jk_*; and *ε_jk_* represents the residual error of the additional unquantified variation between individual samples, which is normally distributed with an average of 0 and standard deviation of *σ_j_*. This model was successfully used in previous studies [4,11,17,24]. 

## 3. Results 

**Figure 2:**
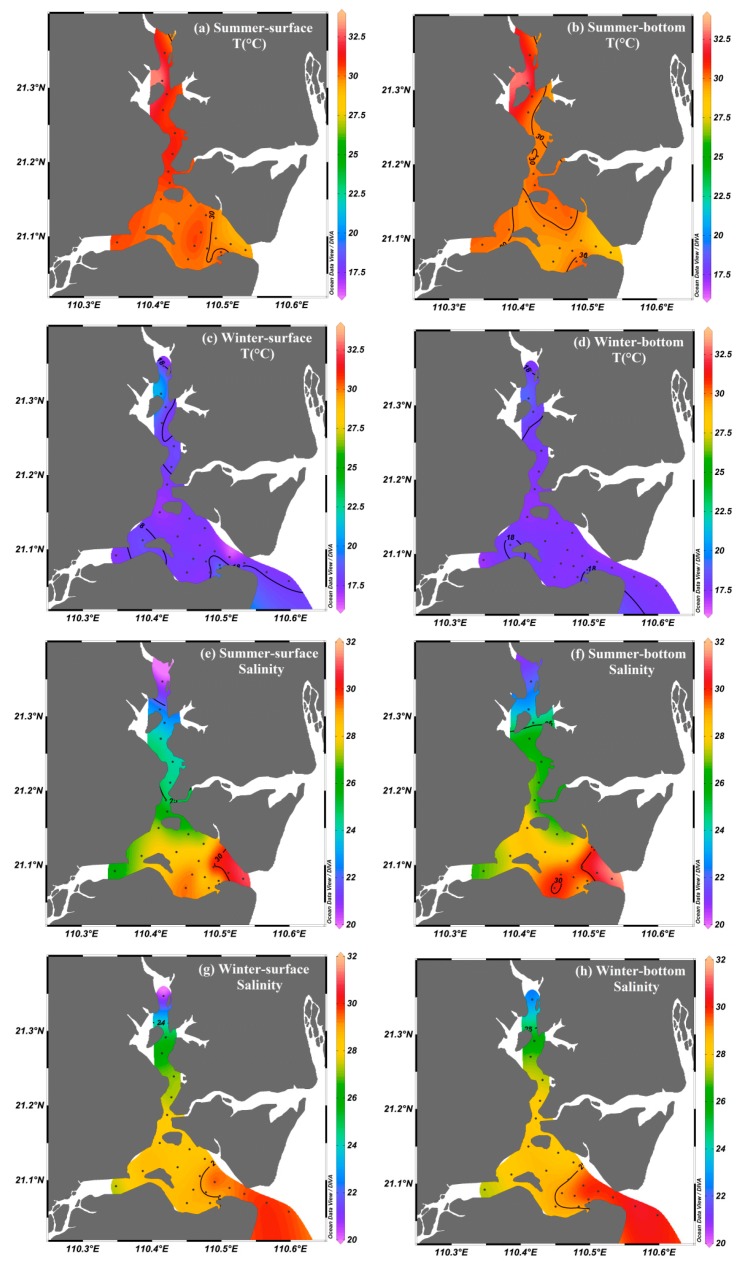
Spatial and seasonal distributions of temperature and salinity in the surface and bottom water of Zhanjiang Bay.

**Figure 3:**
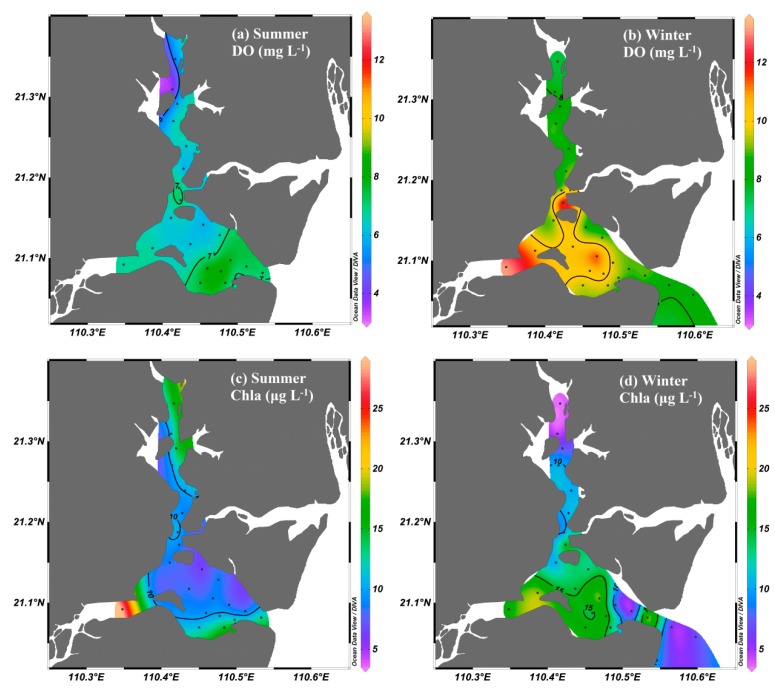
Spatial and seasonal distributions of dissolved oxygen (DO) and chlorophyll *a* (Chl *a*) in Zhanjiang Bay.

The distributions of the physicochemical parameters are shown in Figure 2. The water temperature was lower in winter (16.42–21.26 °C) and higher in summer (28.89–33.44 °C). The spatial distributions of temperature were similar throughout the entire bay area during each season. Salinity increased seaward from 20.02 in the upper bay to >30.00 in the bay mouth during the two seasons. The salinity in the upper bay during summer was lower than that in winter, which may have been influenced by the heavily diluted water during the rainy season. However, the water column was well-mixed in the bay during the sampling periods, and thus exhibited similar values of temperature and salinity in the surface and bottom water (Figure 2). The spatial and seasonal distributions of dissolved oxygen (DO) and chlorophyll *a* (Chl *a*) were presented in Figure 3. DO levels ranged from 3.17 to 8.92 mg·L^−1^ in summer and 7.50 to 12.60 mg·L^−1^ in winter, with higher values in winter than in summer. The Chl *a* levels ranged from 6.11 to 15.42 μg·L^−1^ in summer and from 3.61 to 19.05 μg·L^−1^ in winter, with an average of 10.00 μg·L^−1^ in summer and 11.87 μg·L^−1^ in winter.

**Figure 4:**
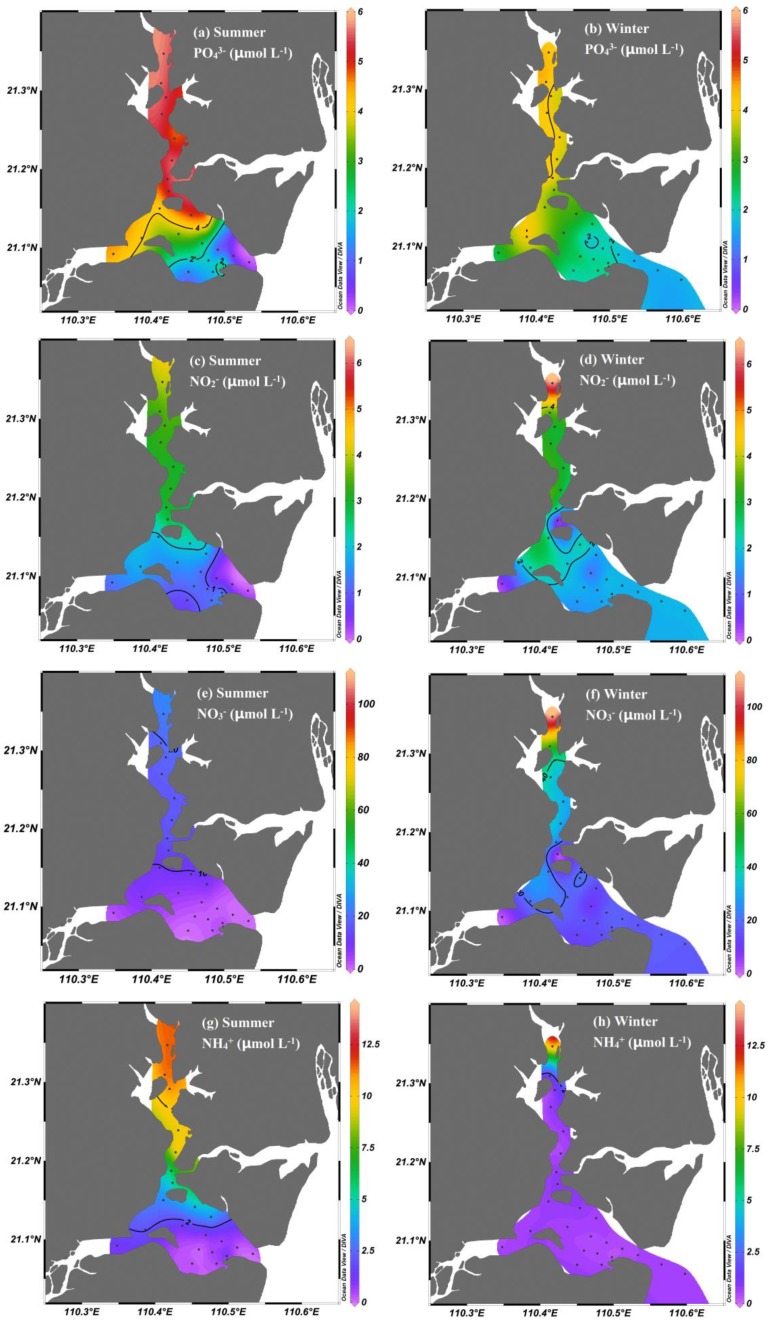
Spatial and seasonal distributions of (**a**) PO_4_^3−^, (**b**) NO_2_^−^, (**c**) NO_3_^−^, and (**d**) NH_4_^+^ in the surface water in Zhanjiang Bay.

The nutrients showed distinct seasonal and spatial variations in the bay (Figure 4). The concentrations of PO_4_^3−^ ranged from 0.13 to 5.68 μmol·L^−1^. In the various forms of inorganic N, the NO_3_^−^ concentration (0.02 to 111.08 μmol·L^−1^, with an average of 16.96 μmol·L^−1^) was the highest, followed by NH_4_^+^ (0.16 to 14.24 μmol·L^−1^, with an average of 2.43 μmol·L^−1^) and NO_2_^−^ (0.08 to 6.40 μmol·L^−1^, with an average of 2.07 μmol·L^−1^). The NO_3_^−^ concentration (with an average of 16.96 μmol·L^−1^) in inorganic N was the highest, followed by NH_4_^+^ (with an average of 2.43 μmol·L^−1^) and NO_2_^−^ (with an average of 2.07 μmol·L^−1^). Generally, the concentration of nutrients decreased seaward, with higher concentrations observed in the upper bay and lower concentrations observed in the bay mouth. Seasonally, higher concentrations of PO_4_^3−^ and NH_4_^+^ were observed in summer, whereas lower concentrations were observed in winter. A significantly high concentration of NH_4_^+^ occurred in winter, except for station Z1 (Figure 4h). A higher concentration of NO_3_^−^ was observed in winter and a lower concentration was observed in the summer. 

**Figure 5:**
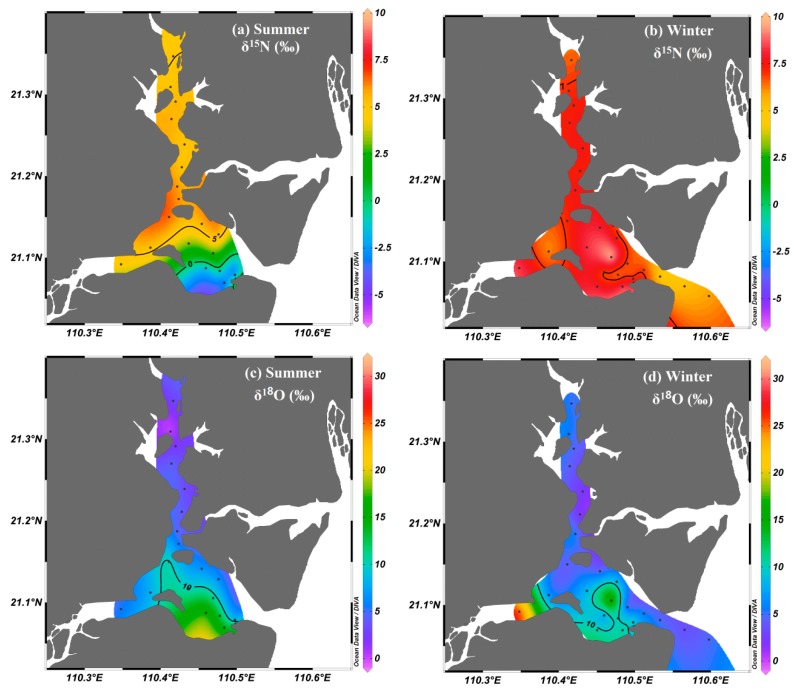
Spatial and seasonal distributions of (**a**) δ^15^N-NO_3_^−^ and (**b**) δ^18^O-NO_3_^−^ in the surface water of Zhanjiang Bay.

The values of δ^15^N-NO_3_^−^ and δ^18^O-NO_3_^−^ ranged from –3.72‰ to 9.66‰ and from –0.30‰ to 17.20‰ with an average of 5.37‰ and 7.15‰, respectively (Figure 5). Higher δ^15^N-NO_3_^−^ values were observed in winter (average of 7.20‰), whereas lower values were observed in summer (average of 3.45‰). Slightly higher δ^18^O-NO_3_^−^ values were found in summer (average of 7.28‰), and lower values were found in winter (average of 7.04‰). There were no significant spatial variations in δ^15^N-NO_3_^−^ values during winter, while the δ^15^N-NO_3_^−^ values in the upper bay were significantly higher than those in the mid-bay and bay mouth during summer. However, the δ^18^O-NO_3_^−^ values increased seaward, and exhibited higher values in the mid-bay and bay mouth (Figure 5c,d). 

## 4. Discussion

### 4.1. Limiting Nutrients in Zhanjiang Bay

Throughout Zhanjiang Bay, the N/P ratios ranged from 0.2 to 30.1, with an average of 6.1, which were significantly lower than the Redfield ratio of 16 (the nutrients at this ratio are utilized by marine phytoplankton) [6]. The high PO_4_^3−^ concentrations (with a minimum value of >5 μmol·L^−1^) in the bay throughout the year suggested that N acts as a limiting nutrient in this ecosystem and that the increase in N is favorable for the proliferation of phytoplankton. Many red tide and algae bloom events were reported in Zhanjiang Bay, which are mainly caused by severe eutrophication [25,26,27]. In addition, the index of nutritional status has increased significantly in past decades, which was caused by the increased amounts of inorganic phosphorus and nitrogen in the coastal seawater of Zhanjiang Bay [25]. Different from the other sub-tropical areas, in which phytoplankton blooms in spring or autumn, the phytoplankton blooms in Zhanjiang Bay occur in summer [27]. This may be related to the large land-based pollution discharge that occurs during the summer in Zhanjiang Bay [19]. Since the highest PO_4_^3−^ level and lowest salinity level appeared at station Z1 (the upper bay), particularly in the rainy season (summer), we speculated that domestic sewage, which contained high levels of PO_4_^3−^, may have been responsible for the elevated levels of PO_4_^3−^. A similar situation has been documented in Xiangshan Bay (China) [6].

Based on the above discussion, N is a limiting nutrient in this bay; thus, it was necessary to study N distribution. The NO_3_^−^ concentration was generally higher during the two seasons in the upper bay than in the higher salinity areas from the mid-bay and bay mouth, which may have been influenced by the oyster breeding process and/or local terrestrial inputs. This pattern of seaward-decreasing NO_3_^−^ was similar to that of NO_3_^−^ distributions seen in other coastal areas. For example, in the Pearl River Estuary (China) [5,28], San Francisco Bay (US) [29], and Elbe Estuary (Germany) [30], higher NO_3_^−^ concentrations occur in the upper bay stretches with a maximum value of >100 μmol·L^−1^, and NO_3_^−^ mainly originates from local river inputs. The significantly high concentrations of NO_2_^−^, NO_3_^−^, and NH_4_^+^ only occurred at station Z1, which may have been influenced by the local contaminant discharge. This area is influenced by frequent and intensive human activities, such as mariculture, industry, agriculture, and shipping. However, the NO_3_^−^ concentrations in the seawater of the bay during winter were higher than those found during summer. We speculated that the heavy runoff during the wet season may transport more nutrients into the inner bay, which is simultaneously influenced by the diluted water from the outer seawater, thereby causing the nutrient concentrations to exhibit a seaward decrease. This was supported by the seasonal variations in salinity in the upper bay, which exhibited a lower value in summer. 

### 4.2. Biological Processes of NO_3_^−^ in Zhanjiang Bay

Generally, biological processes such as assimilation and denitrification can cause isotope fractionation owing to preferential uptake of lighter isotopes (^14^N and ^16^O) [9,13]. If biological processing of NO_3_^−^ occurred, the NO_3_^−^ isotopes would be changed and information on the NO_3_^−^ source would be obscured. Therefore, to reveal the sources of NO_3_^−^, it was important to examine the biological processes of NO_3_^−^ in Zhanjiang Bay. 

In winter, the δ^15^N-NO_3_^−^ values were higher than those in summer (Figure 5a,b). NO_3_^−^ assimilation and microbial denitrification are usually considered likely processes for elevating the isotope values of NO_3_^−^ in aquatic environments, with isotope fractionation factors of 5‰ to 10‰ and 20‰ to 30‰, respectively [5,14,31,32]. In this study, algal assimilation was likely responsible for the increase in δ^15^N-NO_3_^−^ values. NO_3_^−^ assimilation by algae can cause isotopic enrichment of the residual NO_3_^−^, during which, fractionation factors vary among different species [12,33,34]. A winter phytoplankton proliferation was indicated by the Chl *a* levels (with an average of 13.22 μg·L^−1^), and the Chl *a* levels were relatively higher than the levels found in summer (with an average of 9.43 μg·L^−1^). During the winter, the high Chl *a* levels were detected from Z6–Z20 (in the mid-bay and the bay mouth), and, while comparing the lower nutrient concentration in those areas, we considered that the low nutrient concentration may be due to the consumption by the phytoplankton. Along with this phytoplankton proliferation, a pronounced consumption of NH_4_^+^ occurred, owing to the preferential uptake of NH_4_^+^ by phytoplankton. Thus, significantly low NH_4_^+^ concentrations were observed in winter. However, the residual NH_4_^+^ in the seawater was still not completely consumed by phytoplankton, with an average of 0.56 μmol·L^−1^ in the mid-bay and bay mouth. This suggested that the phytoplankton in the seawater would continue to consume NH_4_^+^ in the bay, but not NO_3_^−^ during winter. Thus, the elevated δ^15^N-NO_3_^−^ values in the bay during winter may not have been caused by assimilation. On the other hand, significantly higher levels of DO were observed in the seawater of the mid-bay and bay mouth during winter (range from 8.17 to 13.15 mg·L^−1^, with an average of 9.76 mg·L^−1^); this environmental condition did not seem to favor the process of denitrification in the water. However, we tentatively attributed the δ^15^N-NO_3_^−^ enrichment in the seawater during winter to the interplay between water and sediment. Previous studies suggested that active consumption of NO_3_^−^ in sediments in coastal areas is most likely due to denitrification [4,5,31,35]. Irrigation, biological perturbation, and other physical perturbation can significantly occur in surface sediments, particularly during the winter monsoon period when wind-induced mixing and tidal pumping are strong [5,35]. Such a dynamic environment would result in the bidirectional exchange of nutrients between the sediment pore water and the overlying water, and finally, cause increased δ^15^N-NO_3_^−^ values in the coastal water due to denitrification-induced isotopic enrichment in sediments [5].

However, in summer, NH_4_^+^ and PO_4_^3−^ concentrations decreased gradually from the upper bay to the bay mouth, the concentrations were higher than those in winter, thereby suggesting that the nutrients that originated from the upper bay affected the mid-bay and the bay mouth under the heavy discharge during the wet season. In addition, the decreasing trend of δ^15^N-NO_3_^−^ values (close to 0‰) in summer suggested that assimilation and denitrification were not dominant processes in this season. Moreover, although the concentration of Chl *a* fluctuated from stations Z1 to Z25, there was no clear trend of change in the seawater during summer. This suggested that the seaward decrease in nutrient concentrations was not mainly affected by phytoplankton assimilation, but instead, more likely affected by dilution with the outer seawater. However, relatively high δ^18^O-NO_3_^−^ values and low δ^15^N-NO_3_^−^ values were found in the mid-bay and bay mouth, which may have been influenced by the NO_3_^−^ from atmospheric deposition. The δ^18^O-NO_3_^−^ values were high (>50‰) and δ^15^N-NO_3_^−^ values were low (<0‰) in the coastal area, and some values of dual NO_3_^−^ isotopes in the seawater from the bay during summer were also close to the range of NO_3_^−^ in precipitation [20,36,37]. 

### 4.3. Sources of NO_3_^−^ to the Bay 

**Figure 6:**
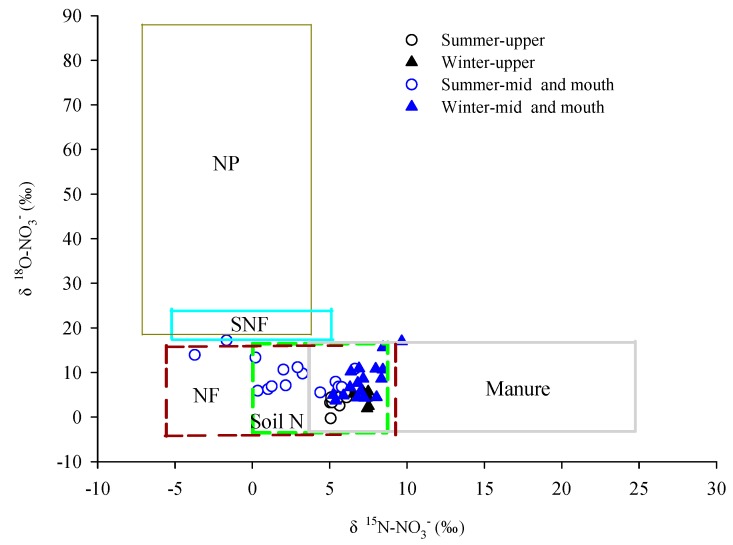
Values of δ^15^N-NO_3_^−^ and δ^18^O-NO_3_^−^ in seawater samples during different seasons in Zhanjiang Bay and various nitrate (NO_3_^−^) sources (boxes). The five potential NO_3_^−^ sources are manure, N fertilizer (NF), soil N [15], synthetic N fertilizer (SNF) [39], and N in precipitation (NP) from the northern area of the South China Sea [37]. The inset in the upper right corner shows an expanded view of δ^15^N-NO_3_^−^ and δ^18^O-NO_3_^−^ values in Zhanjiang Bay during different seasons. The regression was calculated from data for winter in the mid-bay and bay mouth.

Since biogeochemistry cannot account for the variation in δ^15^N-NO_3_^−^ and δ^18^O-NO_3_^−^ in the bay, isotopic characteristics may provide evidence of the mixing from various sources. According to a standard dual-isotope approach [15,37], the range of δ^15^N-NO_3_^−^ and δ^18^O-NO_3_^−^ values in the upper bay during the two seasons suggested that manure, soil N, and N fertilizer (NF) might be the dominant NO_3_^−^ sources for this area (Figure 6). NO_3_^−^ from manure would cause higher δ^15^N-NO_3_^−^ values in the upper bay, and manure has been proposed as a significant NO_3_^−^ source in most coastal areas in China, such as the Pearl River Estuary [5,38] and Xiangshan Bay [6], which may be influenced by local activities. This has caused heavy N inputs to the coastal seawater [3,28]. The mineralization of soil N in the coastal seawater and the underlying sediment pore water have been proposed as important sources of NO_3_^−^ in coastal areas [9,17]. However, in the mid-bay and bay mouth, although the δ^18^O-NO_3_^−^ values were generally higher than those in the upper bay, the ranges of δ^15^N-NO_3_^−^ and δ^18^O-NO_3_^−^ values in the mid-bay and bay mouth were also mostly distributed close to manure, NF, and soil N. These slightly higher δ^18^O-NO_3_^−^ values might have been due to the contribution of NO_3_^−^ from synthetic N fertilizer (SNF) and N in precipitation (NP). However, the contribution of SNF could be eliminated, as it only accounted for <2% of the SNF applied in China [31]. Thus, atmospheric deposition may be one of the sources of NO_3_^−^ in the bay.

To quantify these four NO_3_^−^ sources in the upper bay, the isotope values from manure (12.73 ± 3.4‰ for δ^15^N-NO_3_^−^ and 4.08 ± 0.33‰ for δ^18^O-NO_3_^−^), NF (0.04 ± 1.87‰ for δ^15^N-NO_3_^−^ and 4.08 ± 0.33‰ for δ^18^O-NO_3_^−^), soil N (4.52 ± 2.67‰ for δ^15^N-NO_3_^−^ and 4.08 ± 0.33‰ for δ^18^O-NO_3_^−^) [15], and NP in Zhanjiang Bay (0.80 ± 1.49‰ for δ^15^N-NO_3_^−^ and 52.4 ± 5.07‰ for δ^18^O-NO_3_^−^) [20] were adapted to use the Bayesian mixing model. As discussed above, the nutrients were mainly detained in the upper bay during winter owing to the low discharge found in the dry season. Thus, the NO_3_^−^ sources in the upper bay were quantified during both seasons (summer and winter), while the NO_3_^−^ sources in the mid-bay and bay mouth were only quantified in summer. 

**Figure 7:**
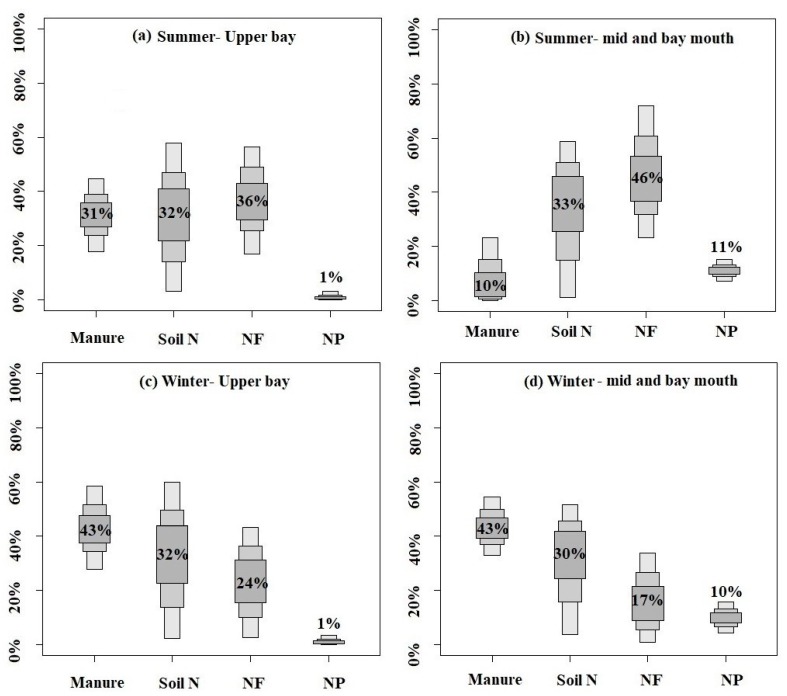
Proportional contributions of the four potential nitrate (NO_3_^−^) sources in Zhanjiang Bay, estimated by the Bayesian isotope mixing model, namely N fertilizer (NF), soil N, manure, and N in precipitation (NP). (**a**) NO_3_^−^ sources in the upper bay during summer, (**b**) NO_3_^−^ sources in the mid-bay and bay mouth during summer, (**c**) NO_3_^−^ sources in the upper bay during winter, and (**d**) NO_3_^−^ sources in the mid-bay and bay mouth during winter.

The proportional contributions of manure, soil N, NF, and NP are presented in Figure 7. In the upper bay, the NO_3_^−^ sources originated from NF (from 17% to 57%, with an average of 36%), soil N (from 3% to 58%, with an average of 32%), and manure (from 18% to 45%, with an average of 31%) during the summer period (Figure 7a), which reflects the local human activities, while the dominant proportional NO_3_^−^ sources mainly originated from manure (from 28% to 58%, with an average of 43%), followed by soil N (from 2% to 60%, with an average of 32%) and NF (from 2% to 43%, with an average of 23%) during the winter period (Figure 7c), indicating the runoff from the upper river basin. The contribution of NP in the upper bay during both seasons was the lowest (1%). In the mid-bay and bay mouth, the dominant proportional contribution of NO_3_^−^ in summer was from NF (with an average of 46%), followed by soil N (with an average of 33%), NP (with an average of 11%), and manure (with an average of 10%) (Figure 7b), which reflects the NO_3_^−^ was mainly sourced from the runoff from the upper river basin. Meanwhile, the highest proportional contribution of NO_3_^−^ in winter was from manure (43%), followed by soil N (30%), NF (17%), and NP (10%) (Figure 7d), which reflects the impact of local human activities. 

The contributions of soil N to NO_3_^−^ in the two seasons were similar, but those of manure, NF, and NP were different. The contribution of manure in winter was significantly higher than that in summer, whereas the contribution of NF in summer was higher than that in winter throughout the bay. During winter (dry season), which was less impacted by the river and rainwater input, the NO_3_^−^ sources were mainly influenced by local activities. By contrast, in the wet season, the heavy rainfall and strong river discharge may have carried more contaminants from the watershed to the bay, thereby changing the local NO_3_^−^ sources. There should be a high contribution of manure in the dry season [31,40]. Thus, the highest NO_3_^−^ concentration in winter may have been influenced by local activities. However, NF, which was mostly in its reduced form, such as urea (71%), NH_4_HCO_3_, and NH_3_Cl (27%), is heavily applied in the catchment agriculture of Guangdong Province [4,5,31] and would be transported to the bay by river discharge and direct discharge from coastal mariculture ponds and wetlands with the heavy rainfall during the wet season. In addition, the heavy rainfall and river discharge could dilute the NO_3_^−^ from manure, and thus, the NO_3_^−^ from manure in winter was found to be significantly higher than that found in summer. These results suggested that runoff exerted an important impact on the shift in NO_3_^−^ sources between the local source and the source from the upper river basin during the two seasons. 

Overall, N input was influenced significantly by the intensive human activities and rapid economic development in Zhanjiang Bay. This influence would be enhanced by the further development of the economic development in Zhanjiang Bay. In addition, global warming seems to cause a higher frequency of hot summers, which favor the proliferation of phytoplankton in the study area. Thus, more attention should be paid to the ecological security of Zhanjiang Bay, and its far-reaching influences on the water environments in the South China Sea need to be considered, and studied, in the future.

## 5. Conclusions

Seasonal values of δ^15^N-NO_3_^−^ and δ^18^O-NO_3_^−^ were measured in Zhanjiang Bay, a closed bay with intensive human activities located in South China, to determine both the predominant sources of NO_3_^−^ and its biological transformation in the bay, as well as the controlling factors influencing the NO_3_^−^ distribution. Significant variations of nutrient concentration were observed in the bay, and the concentration of nutrients decreased seaward, with higher concentrations observed in the upper bay and lower concentrations observed in the bay mouth. High ammonium concentration was found in the bay due to intensive human activities. Relatively low N/P ratios in the bay suggested that nitrogen (N) was a limiting nutrient, which indicates that increasing N was favorable for phytoplankton proliferation. However, less nitrate-based biological processes occurred in the bay, suggesting that the nitrate isotopic characteristics may reveal details of the mixing from various sources. The nitrate sources calculated by the Bayesian mixing model indicated that nitrate sources mainly originated from manure (43%) in the upper bay during the winter, followed by soil N (30%), N fertilizer (17%), and N precipitation (10%), while nitrate sources during summer were mainly N fertilizer (36%), soil N (32%), and manure (31%). This reflects the local human activities in the winter and the runoff from the upper river basin in the summer. Our results suggested that the nitrate dual-isotope method was very useful for tracing the main NO_3_^−^ sources in the condition of the sufficient ammonium, and runoff exerted an important impact on the shift in NO_3_^−^ sources between local source and river basin source during the two seasons in this closed bay.
